# Co-Designing a Digital Intervention for Health Promotion for People With Intellectual Disabilities: Qualitative Study of Team Members’ Reflections

**DOI:** 10.2196/105248

**Published:** 2026-10-16

**Authors:** Ellinor Nilsson, Stefan Johansson, Pontus Henriksson, Kristin Alfredsson Ågren, Ulrika Müssener

**Affiliations:** 1 Department of Health, Medicine, and Caring Sciences Linköping University Linköping, Östergötland Sweden; 2 Clinical Department of Habilitation in Linköping Region Östergötland Linköping, Östergötland Sweden; 3 School of Electrical Engineering and Computer Science Division of Media Technology and Interaction Design KTH Royal Institute of Technology Stockholm, Stockholm Sweden; 4 Department of Design Sciences Lund University Lund, Skåne Sweden

**Keywords:** accessibility, co-design, digital health interventions, health promotion, intellectual disability, participatory research

## Abstract

**Background:**

People with intellectual disabilities experience a disproportionate burden of preventable health conditions, often linked to low levels of physical activity and poor dietary habits. Although digital health interventions hold considerable promise for addressing these challenges, many existing solutions remain difficult to use for this population due to limited accessibility. Co-design is increasingly advocated to develop digital health solutions that better align with users’ needs, yet there is limited empirical guidance for co-design processes with people with intellectual disabilities.

**Objective:**

This study aimed to explore experiences of a co-design process for developing a digital intervention for health promotion for people with intellectual disabilities, from the perspectives of members of the co-design team involved in different roles across the process.

**Methods:**

A qualitative study design was used based on 5 retrospective reflection meetings conducted in southeastern Sweden following a co-design process of 17 workshops. Eight purposively selected members of the multidisciplinary co-design team, including 4 researchers, 2 user experience designers, 1 support worker, and 1 coresearcher with intellectual disability, participated in different constellations. Reflection meetings were held between February and March 2026, and transcribed data were analyzed inductively and interpreted in relation to established co-design frameworks.

**Results:**

The findings reflect the team members’ experiences of the co-design process and point to a number of factors that appeared to support participation and collaboration. These included an extensive preparatory phase focused on recruitment and relationship building, the importance of working concretely and visually already during predesign, and the role of convivial relational practices in supporting confidence, voice, and collaboration. Time demands were mainly related to the iterative and participatory nature of co-design rather than to intellectual disability per se. Ethical considerations related to compensation were also highlighted, particularly in relation to recognizing coresearchers’ contributions on equal terms.

**Conclusions:**

By examining experiences of the co-design process, this study contributes methodological insights into how digital intervention development can be made more accessible, participatory, and responsive to the needs of people with intellectual disabilities. The findings suggest that enabling meaningful co-design with people with intellectual disabilities requires flexible interpretation of existing co-design frameworks, sustained attention to relational collaboration throughout the process while recognizing the ongoing importance of collaboration with support workers, and adaptation of methods rather than a need for development of entirely new ones. Supporting a sense of collaboration and conviviality over time, alongside addressing ethical issues of compensation for coresearchers’ work, may be critical for producing digital interventions for health promotion that are both usable and inclusive.

## Introduction

Digital interventions for health promotion have the potential to reach broad populations and can be adapted to meet diverse needs [1]. To support their relevance and use, involvement of end users is widely emphasized, particularly when targeting individuals with disabilities, including people with intellectual disabilities [2].

Intellectual disability is a neurodevelopmental disorder characterized by significant limitations in intellectual functioning and adaptive behavior with a global prevalence of approximately 1% to 2% [3,4]. The health status of individuals with intellectual disabilities is disproportionately disadvantaged compared with the general population, including a life expectancy up to 20 years less [5,6]. A substantial share of this health burden comes from preventable or treatable conditions [5,7], strongly associated with health behaviors. Previous studies have reported low levels of physical activity [8-10] and poor diet quality [11-13] among people with intellectual disabilities, reflecting disparities in health behaviors and underscoring the importance of finding effective ways to support these behaviors. For people with intellectual disabilities, it is important to recognize the complexity of health literacy when supporting such behaviors due to cognitive and communicative challenges. From a health literacy perspective, these challenges are not only shaped by individual abilities but also by how communication, support, and interventions fit people’s needs and everyday contexts [14].

Over the past decade, digital interventions for health promotion have increasingly been used in the general population, showing promise in supporting healthy behaviors [1], and their use has also grown among people with intellectual disabilities [15,16]. At the same time, effectiveness remains unclear and many solutions are still largely inaccessible [17,18], with systemic barriers across multiple levels limiting opportunities for digital inclusion [19].

Traditional approaches to designing digital interventions, often rooted in user-centered design, tend to verify accessibility and usability late in the development process [20]. Consequently, fundamental barriers frequently remain, resulting in digital interventions that fail to meet accessibility standards and risk excluding users with disabilities [20]. A more inclusive design approach, such as co-design [21,22], which involves the users throughout conceptual and technical development, has the potential to enhance both relevance and accessibility [23]. Co-design emphasizes collaboration and shared decision-making [22] and is increasingly used in health and social care contexts [24] as well as in the development of digital interventions for health promotion [25]. Co-design is defined in a wide range of ways [24] but can broadly be described as “the creativity of designers and people not trained in design working together in the design development process” [22], pointing to a shift in how design processes may be organized. A variety of methods are used in co-design in general, including interviews, workshops, and prototyping activities to explore users’ contexts, generate ideas, and refine solutions, with no single method dominating any phase [25]. Further, co-design can be challenged by issues such as power imbalances, limited time and funding, and difficulties in involving diverse or vulnerable participants [26]. Additional challenges relate to group dynamics and the use of methods that do not always support open and equal participation [26].

Co-design with historically excluded groups, such as people with intellectual disabilities, often calls for extra careful consideration of power dynamics and the use of creative strategies to create conditions for inclusive and safe collaboration [27]. People with intellectual disabilities may experience challenges related to understanding complex language, following abstract reasoning, and engaging in group discussions, all of which are argued to be required in co-design processes [28]. Consequently, research suggests that meaningful participation in co-design processes with people with intellectual disabilities can be supported through specific strategies, including extended time and preparation [29], visual and practical examples [30,31], adapted and personal support [32], and involvement of support workers [31]. However, uncertainties remain regarding both the concept of co-design and how such approaches are applied when working with people with intellectual disabilities [33]. Moreover, relatively little is known about how co-design processes are experienced by people with intellectual disabilities and others involved in such processes [29,33]. As studies labeled as co-design do not always fully reflect participatory approaches [24,34], there is a need for greater methodological transparency in how co-design processes are conducted, particularly when involving people with intellectual disabilities [33]. Understanding how co-design processes are experienced in practice may help researchers and designers organize activities that enable meaningful participation and inform future co-design practices for people with intellectual disabilities, potentially leading to more useful and relevant interventions for this population [35].

The aim of this study was to explore experiences of a co-design process for developing a digital intervention for health promotion for people with intellectual disabilities, from the perspectives of members of the co-design team involved in different roles across the process.

## Methods

### Study Design

The present study is part of a larger research project, Intellectual Disability Integrating Digital Intervention Technology for Health (I DID IT), aiming to promote the health and well-being of people with intellectual disabilities.

This study is based on post–co-design reflection meetings with a purposive sample of co-design participants examining a co-design case involving the development of a digital intervention for health promotion for people with intellectual disabilities. The analytical approach draws on a reflection-on-action practice [36], through which the process was systematically revisited and critically interrogated to inform methodological insights relevant to co-design with people with intellectual disabilities in the development of digital interventions.

The Consolidated Criteria for Reporting Qualitative Research (COREQ) checklist [37] guided the reporting (Multimedia Appendix 1).

### The Case: Co-Design Process

The co-design process explored in this study consisted of 17 workshops aimed at developing the concept for a digital intervention for health promotion for people with intellectual disabilities, covering the first phases of the development. The development was guided by a co-design approach [38], grounded in participatory design principles [39]. Workshop activities were structured in line with the framework for co-design developed by Sanders and Stappers [21], interpreted as a “tangled ball of yarn” illustrating the iterative and nonlinear nature of a co-design process across 4 phases (Figure 1). The framework describes 4 phases of co-design: predesign (understanding current experiences), generative (generating future solutions), evaluative (testing and refining), and postdesign (use and implementation in practice). Although the framework includes evaluative and postdesign phases, the present study focused on the predesign and generative phases, during which the intervention concept was developed.

**Figure 1:**
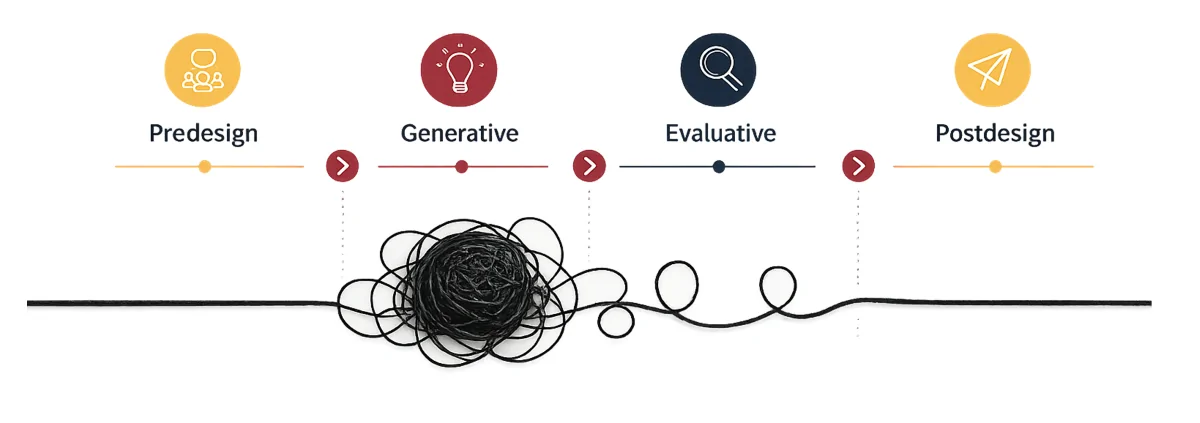
Co-design framework based on Sanders and Stappers [21].

The research team that initiated and planned the workshops consisted of a group with combined expertise in intellectual disabilities, digital health promotion, digital intervention development, digital participation, human-computer interaction, and co-design practices involving people with intellectual disabilities.

In total, 24 coresearchers aged 16 to 40 years with mild to moderate intellectual disabilities participated across different phases of the co-design process, which took place between May 2023 and November 2025 in municipal and community-based settings, as well as at a university, in southeastern and central Sweden. In the predesign phase, 18 coresearchers with intellectual disabilities initially contributed through separate workshops, while in the generative phase, 6 coresearchers with intellectual disabilities participated in a multidisciplinary co-design team involving researchers, user experience (UX) designers, and support workers. One coresearcher with intellectual disability was involved throughout the entire process.

A detailed description of the process, recruitment procedures, workshop structure, and output is provided in Multimedia Appendix 2 and in our earlier published work [40].

### Participants and Data Collection

The primary data for this study consisted of 5 audio-recorded reflection meetings involving members of the multidisciplinary co-design team that took place in February and March 2026 in southeastern Sweden following the completion of the 17 workshops. The co-design process described above and in Multimedia Appendix 2 served as the case reflected upon. Materials used to support and structure the reflection meetings included recruitment materials, planning documents, workshop summaries, field note summaries, and design artifacts from the workshops (Table 1).

**Table 1:** Processes and materials used to support the reflection meetings.

Data source	Description
Recruitment materials	Written study information, consent forms, and a study information video.
Planning documents	Written plans for each workshop, detailing the intended structure, activities, and methods to be used.
Workshop summaries	Summarized accounts of how the workshops were conducted and the methods used.
Field note summaries	Written text by researchers during and immediately after workshops, documenting key discussions and insights, observations about group dynamics, reflections from the facilitators, and comments or informal evaluations provided by participants.
Design artifacts	Images, sketches, sticky notes, and other visual or material outputs created during the workshops. These artifacts illustrated how ideas developed over time and showed the progression toward concept development.

Eight members of the multidisciplinary co-design team were purposively selected to capture experiences from different positions within the process, including planning, recruitment, facilitation, coresearcher participation, and design development. The sample included researchers, UX designers, and coresearchers with professional and lived experience of intellectual disability. The number of participants was determined by the intention to include all key perspectives from members of the co-design team who remained involved in the final workshops, including a coresearcher with intellectual disability who had participated across both the predesign and generative phases. All were approached directly by the first author by email and/or telephone and agreed to participate. The meetings were held with different constellations of participants (Table 2), including 4 researchers: R1, a PhD in occupational therapy with expertise in intellectual disabilities and digital participation; R2, a PhD in human-computer interaction with expertise in cognitive accessibility, universal design, and co-design; R3, a PhD student and physiotherapist working with people with intellectual disabilities; and R4, a PhD in public health and occupational therapist with expertise in digital health interventions and principal investigator of the project. Additional participants included 1 coresearcher with professional experience working with people with intellectual disabilities (CR1), 1 coresearcher with lived experience of intellectual disability (CR2), and 2 UX designers (UX1 and UX2) with experience in digital health applications and administrative systems. The composition of the meetings was guided by the focus of each discussion. Participants were grouped based on their involvement in specific phases and activities of the co-design process to enable reflection on planning and recruitment, workshop implementation, participation experiences, and design development.

**Table 2:** An overview of the reflection meetings.

Reflection meeting	Participants	Location; duration	Focus	Material
1	R1, R2, and R3	Online via Microsoft Teams; 40 minutes	Initial planning and recruitment	Recruitment materials and planning documents
2	R1, R2, and R3	Online via Microsoft Teams; 40 minutes	Workshops 1-3	Workshop summaries
3	R1, R2, R3, and R4	Online via Microsoft Teams; 1 hour 50 minutes	Workshops 4 and 5 and methods used in workshops 1-5	Planning documents, workshop summaries, field note summaries, and design artifacts
4	R3, CR1, and CR2	Daily activity center; 1 hour	Experiences from participating as coresearchers	Workshop summaries and design artifacts
5	R1, R2, R3, R4, UX1, and UX2	Online via Microsoft Teams; 1 hour 30 minutes	Workshops 6-17	Workshop summaries and design artifacts

The first author of this paper, a female PhD student and physiotherapist working with people with intellectual disabilities, was primarily responsible for facilitating the reflection meetings and preparing the materials used in the reflection meetings. Prior to reflection meetings 1-3 and 5, materials from the co-design process were compiled, including recruitment materials, design artifacts, and documented insights. In addition, workshop and field note summaries were developed based on recordings, transcripts, and notes, providing structured accounts of activities, decision-making, and materials used in each session.

The reflection meetings were based on a shared experience of the co-design process in which all participants had been involved, although from different perspectives. The discussions were open and exploratory in nature, while structured around an after-action reflection approach [41]: (1) what was planned and what was the intention with this approach? (2) What actually happened during the workshops and why? (3) Why did the process unfold in this way, including adaptations and collaborative dynamics? and (4) What do these observations imply for co-design with people with intellectual disabilities? Additional prompts to support the reflections addressed decision-making, perspectives from coresearchers, power dynamics, accessibility, and necessary adaptations and were included in the semistructured reflection guide (Multimedia Appendix 3).

For reflection meeting 4, materials were adapted to support the participation of the coresearcher with intellectual disabilities. It followed a more narrative-oriented format supported by visual materials representing different aspects of the process (eg, settings, activities, time structure, and collaboration) alongside design artifacts and photographs from workshops (excluding identifiable individuals) to support recall. Visual scales were used to facilitate expressions and ranking of experiences.

All meetings were audio recorded and transcribed verbatim.

### Data Analysis

Transcripts from the reflection meetings were read several times to gain a sense of the whole. As an initial organizing step, meaning units relevant to the aim were mapped according to the first 2 phases of the co-design framework described by Sanders and Stappers [21]: the predesign phase and the generative phase. Data that did not fit meaningfully within either the predesign or generative phase, but instead cut across both phases, were analyzed separately to capture cross-phase reflections. Within each phase, the data were then analyzed inductively guided by Elo and Kyngäs [42], using Microsoft Excel. The meaning units were coded, and codes with similar content were compared and grouped into categories. In a next step, categories were further abstracted into higher-order categories corresponding to the predesign, generative, and cross-phase aspects of the co-design process. This abstraction involved moving from descriptive categories toward more analytical interpretations of patterns across the material. Table 3 illustrates examples of this process of analysis.

**Table 3:** Examples of the analytic process guided by inductive content analysis [42].

Meaning unit	Co-design phase	Code	Group	Category	Main category
“It is especially important the first time to have a support worker with you”	Predesign	Support worker important in the beginning	Establish a sense of safety early in the process	Creating an accessible starting point for collaboration	Facilitating socially and cognitively accessible entry conditions to the co-design process
“However, we may have been more careful to make sure that these options were always there—that they [the UX designers] produced several alternatives, and then we prioritized.”	Generative	Having several options to prioritize between	Supporting iterative concept development	Guiding iterative design through visual and concrete materials	Involving diverse expertise in iterative and visual teamwork
“When you know each other, you might not need to be afraid of saying something wrong. And that’s also when you might come up with new ideas.”	Cross-phase	Relationships create safety and creativity	Safety	Building relational conditions that support shared ownership and confidence	Enabling equitable and meaningful participation over time

The initial coding and categorization were primarily conducted by EN. Throughout the analysis, categories and emerging interpretations were discussed iteratively with UM and refined through ongoing dialogue. In a later stage of the analysis, the categorization and abstraction were also discussed together with SJ and KAÅ until agreement was reached on the final category structure. These discussions also included reflexive consideration of how the researchers’ involvement in the co-design process and reflection meetings might have influenced the interpretations.

### Ethical Considerations

This research project involving the co-design process examined in this study was approved by the Swedish Ethical Review Authority (Dnr 2022-06682-01 and 2026-00982-02). Participants received information about the reflection meetings and were informed that participation was voluntary and that declining would not affect their continued involvement in the co-design process. Verbal informed consent was obtained before participation in the reflection meetings. Data were handled confidentially and deidentified during analysis and reporting. Potentially identifying information was omitted or generalized. No compensation was provided for participation in the reflection meetings.

## Results

### Presentation of Findings

In line with the analytic approach, findings are presented in relation to the predesign and generative phases of the co-design framework [21], together with cross-phase findings. Within each phase, the analysis resulted in a set of categories capturing aspects of how co-design was experienced, described below and illustrated with representative quotations. An overview of the categories for each phase is presented in Figure 2.

**Figure 2:**
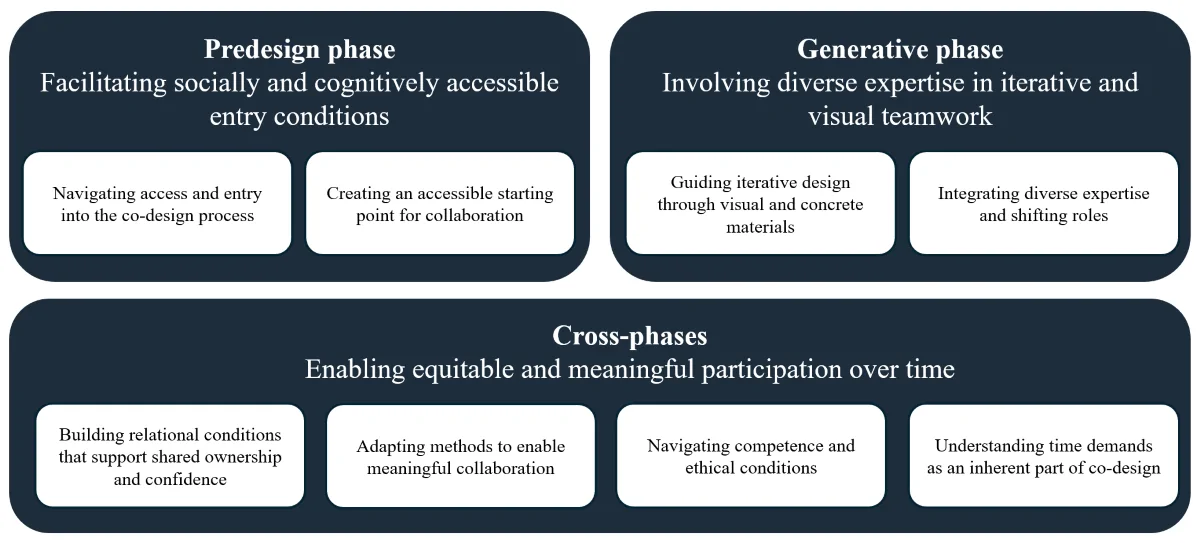
An overview of the categories for each phase of the co-design process.

### Predesign Phase: Facilitating Socially and Cognitively Accessible Entry Conditions

#### Key Reflections on the Predesign Phase

The analysis showed that the predesign phase primarily involved facilitating socially and cognitively accessible pathways into the co-design process, creating conditions that allowed participants to enter and orient themselves within the process. For researchers, this included navigating recruitment and gatekeeping structures, establishing comfort, and planning initial workshops in concrete and understandable ways.

#### Navigating Access and Entry Into the Co-Design Process

Navigating access and entry into the co-design process was described as requiring sustained relational work and engagement with gatekeeping structures for recruitment.

Recruitment constituted a substantial amount of work in what team members describe as a “pre-predesign” phase. Long before the formal co-design activities could begin, researchers needed to invest considerable time in identifying contact routes, visiting services, and establishing trust with key contact persons, including demonstrating both credibility as researchers and relevant expertise in intellectual disability. Previous contacts and the researchers’ prior knowledge of these organizational settings appeared to facilitate this work. The recruitment process did not allow direct access to potential participants with intellectual disabilities; instead, it depended on contact with support workers who knew the individuals well and who were willing to introduce the research team. As one researcher described:

There is almost a pre-pre-design phase where we worked a lot with recruitment. It wouldn’t have been possible without an enthusiastic contact person who could introduce us.

R1

Team members also highlighted that the recruitment video and the easy-to-read study information were helpful tools for supporting this entry work. These materials supported the support worker in approaching potential participants, making the invitation clearer and more accessible.

#### Creating an Accessible Starting Point for Collaboration

Prior to the workshops, efforts were made to identify individual support needs among participants, including aspects related to communication, transport, and access to assistive resources. This resulted in preparatory measures such as sharing images of the workshop environments in advance, which team members experienced as valuable for supporting familiarity and participation.

Team members described how taking a more informal and personal approach in the first meetings appeared to help lower the threshold for participation. In addition, having a support worker, already known to the participants, present during the first sessions contributed to a sense of safety and orientation in a situation that might otherwise feel uncertain: “It is especially important the first time to have a support worker with you” (CR2)*.* Guidance about this role during workshops, such as not speaking on behalf of participants, but supporting communication, memory, and task orientation, was provided. Reflections pointed out that a more structured introduction for support workers could have strengthened the early phase further. When support workers were well oriented, this created conditions in which participants could both feel more at ease and have someone familiar to talk through ideas with: “People need help with making association” (R1)*.* Team members described the role of support workers as uniquely positioned in co-design with people with intellectual disabilities. Support workers both enabled participation, helping with memory, communication, and “thinking aloud,” and participated as stakeholders in the process, which required a careful balance between offering support and contributing to the process without steering the discussion. Because participants did not always control or manage their own schedules or transport, support workers were also crucial for making participation practically possible.

The team also found that concrete activities in the predesign phase played an important role in making work during the predesign phase accessible. Rather than beginning with broad or abstract questions, often used in predesign, this predesign phase started by working on concrete ideas and examples early on. Working with tangible ideas in this way appeared to help participants articulate thoughts that might have been difficult to express in response to open-ended questions alone. The team reflected that these hands-on methods enabled richer discussions and a clearer understanding of participants’ perspectives at this early stage than interviews would likely have produced: “You don’t really learn that much from interviews, and there’s a risk that you think you understand more than you actually do” (R2). Team members noted that, since UX designers were not part of the initial knowledge development workshops, involving them earlier might have streamlined the process by clarifying early information needs.

### Generative Phase: Involving Diverse Expertise in Iterative and Visual Teamwork

#### Key Reflections on the Generative Phase

In the generative phase, the use of visual and concrete materials was described as guiding participants to explore multiple ideas simultaneously and engage in an iterative development of concepts. The findings also pointed to the experience of working in a multidisciplinary team, where balancing responsiveness to participants’ perspectives with professional expertise was important.

#### Guiding Iterative Design Through Visual and Concrete Materials

Visual and concrete materials were described by team members as central to the generative work, enabling the team to explore several design directions in parallel. Rather than converging early, the team described how they intentionally kept multiple alternatives open and revisited them repeatedly, adjusting concepts as new workshop insights emerged. Instead of starting from blank pages, participants were invited to engage with concrete alternatives prepared by the UX designers, which were grounded in insights from earlier workshops. UX designers were tasked with preparing multiple proposals for each design element that could be compared, ranked on a scale, and discussed side by side. The ranking scale was perceived as supporting participants to consider multiple alternatives simultaneously, supporting comparative reasoning and elevating the discussion. When participants sometimes held differing views, with similar elements placed differently, the discussion on their placement on the scale was described as helping to make shared priorities visible and contributing to consensus-based decisions.

Simple sketches of app screens together with concrete examples of potential features, such as specific functions the app could include, supported participants in understanding options and reacting to them in concrete ways. Hands-on activities, such as being able to move, modify, or compare visual elements, helped participants articulate preferences that team members felt would not have emerged through discussion alone. For the UX designers, this approach required preparing more visual material and more versions of each idea than in conventional development: “We worked with more visual material than we usually do, and preparing it definitely takes more time” (UX2). Although time-consuming, working with several parallel concepts was described as essential for keeping the evolving design directions close to participants’ perspectives rather than being shaped primarily by professional assumptions. This way of working also required greater visual clarity and more straightforward language than UX designers were used to but was described as potentially strengthening design work in other projects as well.

#### Integrating Diverse Expertise and Shifting Roles

The generative phase required an active interplay between professional and lived-experience expertise, with roles shifting as the work unfolded. UX designers, researchers, support persons, and coresearchers with lived experience of intellectual disabilities contributed from their respective perspectives. Expertise was used fluidly rather than through fixed role boundaries. The fact that team members, including UX designers, engaged as participants in the process was experienced as contributing to a more integrated collaboration across different forms of expertise, rather than a distinction between professionals and users. This was experienced as somewhat challenging as roles shifted over time, with UX designers moving from participating alongside others to gradually facilitating the workshops, requiring a constant balance between staying engaged as a participant and knowing when to contribute design expertise: “I sometimes felt afraid of taking over, that if I shared my opinions, they might just follow along” (UX1).

UX designers described that they worked in a more participatory and immediate way than in conventional processes, developing ideas together with the team rather than collecting workshop data and later returning to interpret it at a distance:

We actually get to know the target group and work with them directly, and bounce ideas back and forth. That gives us completely different possibilities for thinking about things [...] we have much better conditions for creating something that works well.

UX2

### Cross-Phases: Enabling Equitable and Meaningful Participation Over Time

#### Key Cross-Phase Reflections

The analysis identified that enabling equitable and meaningful participation was an ongoing effort across the co-design process. This was described as an overarching process that cut across both the predesign and generative phases and involved continuous efforts to establish appropriate conditions, foster trust and relational aspects, and adapt methods and communication.

#### Building Relational Conditions That Support Shared Ownership and Confidence

Patterns of comfort, familiarity, and collegial interaction gradually developed as the group worked together across sessions. While some relational elements were intentionally included in the workshop design, much of the relational development took shape through small moments such as traveling together, waiting for others to arrive, informal conversations before and after workshops, sharing meals, and having contact between workshops. This was described as contributing to a sense of familiarity that was difficult to achieve through structured activities alone. As relationships took shape, participants with intellectual disabilities were described as participating more actively, contributing ideas, and responding to others’ suggestions in ways that indicated growing ease and confidence: “Now it feels like we know each other much more...when we started, you didn’t really know anyone.” (CR2), “Do you think we would have come up with the same ideas if we didn’t know each other?” (R3), “I don’t think so. Not in the same way.” (CR2). This also included a greater willingness to express critique and alternative views, which was interpreted as an indication of comfort in voicing opinions that differed from those of the group. Team members reflected that there could have been value in strengthening the informal connections further, for example, through a shared group chat rather than individual messaging:

And sometimes I think, oh, it would have been great if we’d had a group chat where we were all included, so you could just say, like, ‘How is it again? It is tomorrow, right?’

CR1

Group composition was important for how relationships and comfort developed across the process. In the predesign phase, participants with intellectual disabilities were recruited from the same service, already knowing each other, and met in their own familiar setting, a combination that was described as creating safety and supporting the first steps into the collaboration. At the same time, team members noted that existing relationships could influence dynamics, for example, if individuals preferred not to take part together. Entering the generative phase, the group shifted from being a familiar and homogeneous constellation to a more mixed team, and workshops were held at the university, making the setting less familiar for participants with intellectual disabilities. In this context, comfort was supported by the fact that the facilitator had already met all participants in their own setting before the first workshop in the generative phase, together with having a known support worker present. At the same time, team members reflected that holding the workshops at the university carried a meaningful value of its own, beyond practical considerations:

I get the sense that there is a value in us being there, in that you feel more important. Now we are in a real workplace where people have meetings in dedicated meeting rooms. Even if it might have been a bit scary, I get the impression that they liked it.

R2

Team members also reflected that an informal study visit to the university before beginning workshops there would likely have made the situation less demanding for participants.

Team members reflected that managing power dynamics within the co-design team required intentionally presenting the group as a set of participants with different kinds of expertise rather than as a hierarchy of professionals and nonprofessionals. This included seemingly minor choices, such as how researchers introduced themselves or dressed, which helped shift attention away from professional status and toward the shared work: “I don’t need to present myself through my job titles, but more in terms of me as a person and my interests” (R3). Team members also emphasized that balancing power depended on having enough coresearchers with lived experience, as they were seen as the most important contributors for grounding the work. With too few, the collaboration would risk leaning toward professional perspectives, while a more balanced mix helped support shared ownership.

#### Adapting Methods to Enable Meaningful Collaboration

Practical, communicative, and methodological adaptations were highlighted as playing an important role of the co-design process. The team’s reflection suggested that these adaptations, ranging from the use of easy language to the use of hands-on and concrete materials that supported discussion within the team, were essential for enabling participants to understand, engage with, and influence the design activities, supporting shared decision-making.

Communication needed continuous adaptation across the process. Participants often formed thoughts while talking, which made it important to allow detours and side paths in discussions, as “useful insights often came in the rambling” (R2). Visuals and concrete elements were perceived to help participants with intellectual disabilities anchor their thinking when open questions became too abstract. For example, being able to rank and contrast visual representations of different app ideas on a scale appeared to support more nuanced discussions about what worked well, what was less suitable, and why. Either-or or gently leading questions were often necessary to support expression, and small prompts or playful tricks could help get conversations started. Small-group discussions worked better than whole-group or individual tasks because having a few people to talk with made it easier for participants to develop and build on their ideas: “It becomes quite messy when there are too many of us [...] we work better when we divide us into smaller groups instead” (CR2). Across sessions, supporting participants in “thinking aloud” by talking to someone else was described as essential for enabling meaningful participation.

Methodological adjustments were a continuous part of the co-design work. Much of this was centered on preparing activities and materials that were concrete and easy to engage with, such as ranking alternatives, drawing, or working with examples that provided something to react to. Meeting several times was essential not only for generating insights but also for learning how the group worked and adapting the workshop materials accordingly.

#### Navigating Competence and Ethical Conditions

The co-design process also challenged the research team to navigate a set of ethical and competence-related conditions. Experience and knowledge of intellectual disability were described as essential for setting the scene for the work, shaping how sessions were framed and facilitated, and attending to participants’ needs as they arose. Being flexible, attentive, and creative as a workshop facilitator also required knowing how to support conversations in ways that were accessible and manageable for people with intellectual disabilities. This specific expertise was especially important in the early stages, when sessions were primarily led by researchers with experience of intellectual disability, while the UX designer initially participated alongside others rather than taking on a leading role. By not leading from the outset, UX designers had the opportunity to observe and learn how the group worked and communicated. This understanding made it easier to later step into a more guiding role with a clearer sense of how to frame activities and communicate in ways that best supported participants.

Compensation was an important research prerequisite for participation from the researchers’ point of view. Payment was considered ethically necessary once the work took the form of a collaborative multiexpertise team in the generative phase, where coresearchers contributed labor comparable to that of other members of the group: “I feel that it’s ethically right, and that they’re not really participants in the research but our colleagues” (R4). This differed from the predesign phase, where participants were treated more as research participants and therefore received a gift card rather than hourly payment. The distinction between being a research participant in predesign and a paid coresearcher in the generative phase clarified expectations. The hourly compensation in the generative phase was considerably higher than what coresearchers typically earned in their daily activity service, which raised ethical reflections within the research team and prompted contact with participants’ legal guardians to ensure that the compensation would not negatively affect other forms of benefits. However, the compensation was not perceived to influence motivation, as 2 participants chose to withdraw despite the payment.

#### Understanding Time Demands as an Inherent Part of Co-Design

Time demands in this co-design process were described as stemming mainly from the iterative and evolving nature of the work itself, even though the earliest stages of the project required additional time to establish conditions for participation for people with intellectual disabilities:

I definitely think the process could have taken less time overall than it ended up doing. The time aspect wasn’t primarily affected by people with intellectual disabilities.

R3

The process required continuous follow-up between sessions, repeated cycles of refinement, and ongoing communication and coordination, all of which contributed to the overall duration. Time was seen as a central resource that allowed ideas to mature, relationships to form, and insights to be revisited, all of which were said to strengthen the quality of the collaboration. Yet, reflections suggested that the overall time frame could have been shorter with a clearer overarching structure and more process experience within the team, while still preserving the flexibility that co-design depends on.

## Discussion

### Principal Findings

This study explored experiences from a co-design process involving the development of a digital intervention for health promotion with people with intellectual disabilities.

The findings indicate that the co-design process with people with intellectual disabilities involved an extensive preparatory phase requiring substantial work and suggest that enabling participation begins well before collaborative design activities commence. Recruitment did not involve direct access to potential participants. Instead, access was preceded by an initial assessment carried out by others, such as support workers or managers, who evaluated whether the project might be appropriate for a particular person. Here, gatekeeping was understood as both facilitating access and potentially limiting who was invited to participate, as support workers could help build trust and ensure appropriate support while also shaping access to the project. This may have limited access for people with intellectual disabilities who are not already embedded in service-based contexts. This aligns with previous research [43] showing that gatekeeping practices are shaped by support workers’ attitudes, knowledge, and organizational conditions, and that access to research is often mediated by relational and contextual factors rather than direct participant choice. Recruitment therefore places particular demands on the research team, including prior knowledge of intellectual disability, familiarity with organizational contexts, and the ability to develop recruitment materials understandable and accessible to both gatekeepers and potential participants. The need to clarify the role of support workers was not limited to recruitment but also applied during the workshops themselves. Previous research has similarly emphasized that support workers need clear information about their role in co-design activities [31]. In the present study, support workers were found to be important for supporting memory, communication, orientation, and practical participation, but their role needed to be framed carefully to avoid unintentionally filtering or steering participants’ contributions.

From a theoretical perspective, the “preparatory phase” resonates with, but also extends, the co-design framework by Sanders and Stappers [21]. Although the framework visually suggests activity taking place before the formal predesign phase, the boundaries of this stage appeared less linear when co-designing with people with intellectual disabilities. Rather than a brief preparatory moment, this early phase involved ongoing learning and adaptation from the researchers’ point of view, during which decisions about when, where, and how to initiate workshops were shaped through dialogue with support workers. This finding resonates with previous co-design literature emphasizing preparatory work as a distinct and important part of co-design, including activities such as defining roles, preparing facilitation approaches, and establishing conditions for participation before design activities begin [44]. In co-design with people with intellectual disabilities, being attentive to this early, largely invisible phase may be particularly important, as it is central to creating the right conditions for engagement and supporting genuine participation [45].

Relationship-building is widely recognized as a core component of successful co-design processes, regardless of participant group, as it shapes trust and collaboration and mediates power imbalances [46-48]. In this context, relational efforts to foster togetherness and creative interaction can be understood through the concept of conviviality, defined as autonomous and creative interaction among people and their environment [49] and highlighted as a key quality in co-design [50]. Our findings, aligning with earlier studies [32], suggest that such relational work may be even more important when co-designing with people with intellectual disabilities. Previous research has shown that people with intellectual disabilities may be inclined to provide affirmative or agreeable responses, particularly in unfamiliar or hierarchical contexts [51]. In this study, what could be interpreted as conviviality appeared to be associated not only with a greater willingness to express oneself openly but also with the ability to voice critique and disagreement, also found in other co-design research [31]. The findings indicate that conviviality was shaped to a considerable extent through low-threshold interactions that occurred around the formal workshops, fostering not only familiarity but also a sense of shared belonging among participants, while also illustrating how the role of support workers may at times constrain the development of direct convivial relations between participants. This suggests that fostering convivial collaboration may require a gradual shift from reliance on intermediary support toward direct relational engagement once participation has been established.

Although systematic evidence remains limited [52,53], simplified language and visual support are commonly used within accessible communication practices for people with intellectual disabilities. Research suggests that such support may help people express opinions and preferences when used flexibly and adapted to individual communication needs and preferences [54]. In this study, the value of using pictorial support not only for comprehension but also for comparison of different options and evaluation was highlighted, preferably in small-group discussions. From a design perspective, this made value judgments and trade-offs more visible and discussable, as ideas were considered in relation to one another rather than in isolation.

Building on this, the findings further suggest that working in a concrete and idea-based way already in the predesign phase may be particularly important when co-designing with people with intellectual disabilities. In the framework by Sanders and Stappers [21], predesign is primarily described as a phase for understanding people’s experiences in the context of their lives, building empathy, and preparing participants for later co-design activities. In the present study, participants were introduced early to design-oriented activities, with the intention of creating empathy through direct engagement with ideas rather than through abstract discussion. Activities commonly associated with the generative stage, with more focus on exploring ideas and design opportunities together [21], were used already during the predesign workshops and functioned as a means of understanding participants’ perspectives at an early stage. Rather than bypassing empathy-building, working with concrete and speculative idea generation seemed to provide an accessible way for the workshop participants to articulate experience, need, and desire that presumably might have been difficult to express through open-ended questioning alone, in line with previous findings [30,31,55]. These findings suggest that, in co-design with people with intellectual disabilities, methods typically located in the generative phase may productively be moved into predesign. Rather than viewing this as a deviation from established frameworks, it may be understood as an adaptation that aligns the design process more closely with participants’ communicative and cognitive strengths.

Co-design processes are often portrayed as time- and resource-intensive across contexts [23,33,56], also noted in this study. However, the findings suggest that time demands were not primarily related to working with people with intellectual disabilities, but rather to the nature of co-design and the research process itself, as also reported in previous disability research [57]. While additional time was needed in early phases, particularly for recruitment and preparation to enable participation, the design activities themselves did not appear to require more time than anticipated. At the same time, reflections within the team suggest that the overall duration of the process might have been reduced through further refinement of the research process, including a clearer overarching structure. Importantly, time was not experienced solely as a constraint, but as a resource that supported reflection, iteration, and a deeper understanding of participants’ perspectives. Seen in relation to research showing that many digital health solutions fail to meet accessibility needs [20], the time devoted to participatory and visually grounded design work may instead represent a responsible use of resources.

Recognizing the work of coresearchers with lived experience through compensation is an important part of co-design [58,59] and is pointed out as an important ethical consideration also in this co-design process. This is consistent with international expectations in patient and public involvement and participatory research, where contributors with lived experience are increasingly expected to be recognized and compensated for their time and expertise [60]. While not problematic in this study, compensation required careful coordination to ensure it aligned with participants’ financial situations and did not negatively affect their access to welfare benefits, a complexity of compensation in participatory research noted in previous literature [61]. This points to a broader ethical and structural tension. While funding bodies and journals increasingly encourage or require the involvement of people with lived experience as coresearchers, existing ethical review processes might not be well aligned with compensating coresearchers as paid contributors rather than as research participants receiving minor compensation. If these issues are not addressed, there is a risk that coresearchers with lived experience are either compensated at levels that do not reflect their contributions, despite performing comparable work to other team members, or that receiving wages creates personal or financial difficulties. From this perspective, questions of compensation should be recognized not only as a project-level ethical issue but also addressed as a systemic challenge for inclusive research practices.

### Implications for Co-Design Practices

The implications of this study are primarily methodological and draw on the participating team members’ reflections on how co-design of digital interventions can be organized to support accessibility, usability, and meaningful participation when working with people with intellectual disabilities. Based on the findings, several practical implications (Textbox 1) can be proposed for how both research and practice can be planned and conducted when involving people with intellectual disabilities. Figure 3 illustrates the findings of this study, interpreted in relation to the co-design framework by Sanders and Stappers [21].

**Practical implications for co-designing digital interventions with people with intellectual disabilities.**

**Plan for an extended preparatory phase**

- Substantial preparatory work is required before the predesign phase, including recruitment through organizational contexts and identification of individual support needs (eg, transport, environment, and communication), with support workers facilitating participation.

**Assemble a well-balanced design team early**

- Early involvement of designers, together with a sufficient number of coresearchers with lived experience and support workers, supports continuity, balanced perspectives, and collaboration over time, with attention to valuing different forms of expertise.

**Facilitate with shifting expertise**

- Facilitation should be informed by expertise in intellectual disability in the early phases, with a gradual shift toward design expertise and co-facilitation in later phases of the process.

**Foster convivial collaboration through interaction in everyday life**

- Support workers are essential early on, but over time, direct communication and relationships within the design team should be supported where possible.

**Use visual and concrete methods (adapted for participants’ needs)**

- Simplified language and visual support should be used not only for comprehension but also to enable ranking and support iterative discussions of multiple ideas and options. Creative, idea-based activities already in the predesign phase support understanding and stimulate discussion.

**Plan for the whole process with time as an investment**

- Additional time is mainly required for recruitment and preparation and should be seen as integral to supporting an inclusive co-design process, while a clear plan for the overall process may help minimize avoidable time delays.

**Compensation reflecting contribution**

- Compensation for coresearchers should reflect their contributions, while accounting for how payment interacts with benefits and ethical review processes.

**Figure 3:**
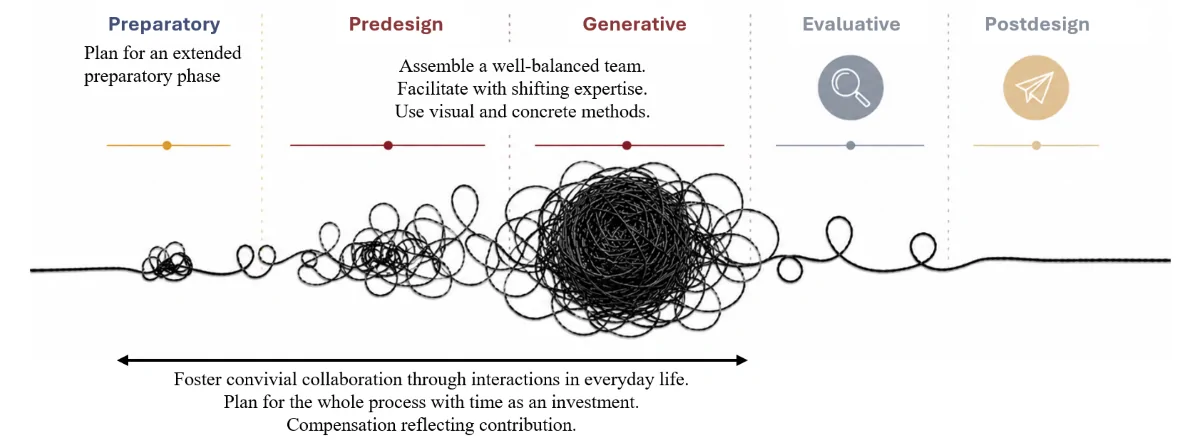
Findings of this study interpreted in relation to the co-design framework by Sanders and Stappers [21], illustrating key considerations across phases when co-designing digital interventions with people with intellectual disabilities.

### Study Limitations

This study is not without limitations. Although the reflection meetings included different perspectives from the multidisciplinary co-design team, only one coresearcher with lived experience of intellectual disability participated in the reflection meetings. The limited inclusion of lived-experience perspectives therefore restricts the extent to which the findings can reflect the diversity of experiences among participants with intellectual disabilities. Furthermore, as the meetings were conducted retrospectively, the findings may have been influenced by participants’ interpretations of the process and selective recall of events. At the same time, considering the focused aim on a specific case, the study was considered to have sufficient information power [62], as key perspectives were represented across the reflection meetings. In addition, participants reflected on a range of materials from the co-design process, including workshop summaries, field notes, planning documents, and design artifacts, which supported recall and helped mitigate retrospective bias. Trustworthiness was further supported through several measures guided by Kyngäs et al [63], including the involvement of researchers with different disciplinary backgrounds in discussing and refining interpretations, and transparent documentation of the analytical process.

A key methodological consideration concerns the researchers’ dual role as both participants in the reflection meetings and analysts of the material, raising concerns regarding the confirmability of the study. While this perspective provided rich contextual knowledge and supported nuanced interpretation, it also required reflexive attention to potential bias. Reflexive discussions within the research team contributed to confirmability by making it possible to question and make explicit how interpretations were developed. However, no external researcher reviewed the coding, and the generally positive reflections on how the process functioned may partly reflect the team’s investment in the work and the collaborative nature of the reflection meetings, which may have encouraged socially desirable responses. Involving an external facilitator in the reflection meetings or an external reviewer of the coding could have further strengthened confirmability. At the same time, the findings may also reflect the research team’s substantial experience in co-design and in working with people with intellectual disabilities, which likely helped anticipate and address potential challenges in advance.

Further, while the findings reflect a single, context-specific case, transferability was supported by a thorough description of the co-design process, together with interpretation of the results in relation to an established co-design framework.

### Conclusions

Drawing on experiences from a multidisciplinary design team working collaboratively to develop the concept for a digital intervention for health promotion, this study found key considerations for co-design with people with intellectual disabilities, interpreted in relation to an established framework. The primary contribution of this study is methodological, offering insights into how co-design processes can be organized to support meaningful participation in digital intervention development. Rather than relying on preparatory user research conducted at a distance, meaningful co-design can be enabled through careful preparation, knowledge of the target group, idea-based activities early on, and sustained direct collaboration with participants throughout the process. When co-design processes are intentionally conducted, people with intellectual disabilities can gain meaningful access to the design arena. These methodological insights may support future co-design processes in which accessibility and usability are considered from the earliest stages of digital health intervention development, with the potential to contribute to more equitable access to health. As this study focused on the predesign and generative phases, continued involvement of people with intellectual disabilities will be important in later technical development, evaluation, and implementation to assess whether the intervention concept translates into practice as intended.

## Appendix

Multimedia Appendix 1 COREQ (Consolidated Criteria for Reporting Qualitative Research) 32-item checklist.

Multimedia Appendix 2 Co-design process, recruitment procedures, workshop structure, and output.

Multimedia Appendix 3 The semistructured reflection guide.

## Abbreviations

COREQ: Consolidated Criteria for Reporting Qualitative Research
I DID IT: Intellectual Disability Integrating Digital Intervention Technology for Health
UX: user experience

## Data Availability

The data that support the findings of this study are available from the corresponding author upon reasonable request.
