# Supplementary material for: Co-Designing a Digital Intervention for Health Promotion for People With Intellectual Disabilities: Qualitative Study of Team Members’ Reflections
Source: J Med Internet Res. 2026 Oct 16;28:e105248. doi: 10.2196/105248 (PMC13722925; doi:10.2196/105248)
Supplement: Supplementary file 2 [file jmir_v28i1e105248_app2.docx]

## The Co-Design Case

The co-design case explored in this study consisted of 17 workshops conducted between May 2023 and November 2025 in southeastern and central Sweden aimed at developing a digital intervention for health promotion for people with intellectual disabilities. The development of the digital intervention was guided by a co-design approach [38], grounded in participatory design principles [39]. The research team that initiated and planned the workshops consisted of a group with combined expertise in intellectual disability, digital health promotion, digital intervention development, digital participation, human–computer interaction, and co‑design practices involving people with intellectual disabilities. The workshops were structured in line with Sanders and Stappers’ co-design framework [21], which describes an iterative process across predesign, generative, evaluative, and postdesign phases. The process described here primarily covered the predesign and generative phases and focused on developing an intervention concept rather than a fully developed product.

Drawing on the research team’s prior experience, the co-design process was carefully planned to support meaningful participation among participants with intellectual disabilities and to create conditions for contributions on more equal footing with support workers, designers, and researchers. Workshops were conducted in familiar and accessible environments intended to promote comfort and included elements such as shared meals to facilitate informal interaction and support a collaborative atmosphere. In preparation for each new group of workshops, efforts were made to identify participants’ specific support needs, including aspects related to transport, communication, breaks, and other practical or environmental adjustments.

### Participants and Recruitment

To involve a broad range of stakeholders with relevant experience of intellectual disability, recruitment for the workshops followed a purposive sampling strategy. Key contact persons within existing networks in municipal services for people with intellectual disabilities and disability organizations in southeastern and central Sweden were engaged to distribute study information to potential participants and support informed decision making. Study information was provided through easy-to-read written materials and an informational video, which contact persons used to introduce and explain the study. Interested individuals were subsequently offered further information through visits by researchers to community-based services, also providing an opportunity to establish an initial relationship with the research team.

Inclusion criteria for participants with intellectual disabilities were: (1) age 16–40 years, (2) mild to moderate intellectual disability, (3) ability to speak Swedish, and (4) ability to understand written and/or verbal information in short, easy‑to‑read Swedish.

Recruitment resulted in three groups of participants with intellectual disabilities involved at different stages of the co-design process. The first group consisted of eight participants recruited from a leisure activity center, together with one support worker from the same setting (workshops 1–3). The second group included ten participants attending a folk high school, accompanied by five support workers (workshops 4–5). The third group was a multidisciplinary co-design team, consisting of six participants with intellectual disabilities, two UX designers, three support workers/professionals, and three researchers collaborating more intensively in the remaining workshops.

As part of the overall intervention development, separate workshops were also conducted with other stakeholders, including professionals and relatives. These workshops are not included in the present study, as the focus was on co-design practices involving people with intellectual disabilities. Findings from workshops with professionals and relatives contributed complementary insights to the development of the intervention, together with the first five workshops involving people with intellectual disabilities, as reported elsewhere [40].

### Concept Development Process

The early predesign workshops focused on exploring participants’ experiences, needs, and ideas related to health promotion, as reported in our earlier work on the core qualities of the intervention [40]. Insights from these workshops were subsequently brought into the multidisciplinary co-design team and used as a basis for concept development. Between workshops, researchers and/or UX designers summarized insights, design ideas, and points requiring further exploration. These summaries were then brought back to the following workshops to allow the team to review, confirm, adjust, or further develop the ideas.

As the process progressed, UX designers translated ideas generated during the workshops into visual materials, including screen mock-ups and alternative concept suggestions. These materials were used in later workshops to support discussion, comparison, and prioritization. Decision-making was conducted collaboratively during the workshops, often through small-group activities in which participants ranked, commented on, or selected between different ideas and options. In some cases, UX designers merged ideas or design directions to accommodate several needs or preferences that had been prioritized by the group. In this way, decisions were not made outside the workshops by researchers or designers alone but were continuously revisited and negotiated with the wider co-design team.

Through this iterative process, the co-design team developed a concept for a game-based smartphone application structured around a recurring “weekly mystery” that users could solve individually or together with others. Challenges related to physical activity and healthy eating formed key components of the application. An overview of all workshops involving participants with intellectual disabilities during the co-design process, including focus, methods, and key insights, is presented in Table 1.

**Table 1.** Overview of the workshops conducted with people with intellectual disabilities during the co-design process.

|  | **Workshop**  **(week; duration)** | **Participants** | **Venue** | **Focus** | **Methods** | **Key insights** |
| --- | --- | --- | --- | --- | --- | --- |
| **Group 1** | 1  (1;2 h) | 7 people with ID^a^  1 support worker  3 researchers | Leisure activity center | - Establish relation - Describe the aim of the project - Develop knowledge of health: What is health for you? | - Joint meal, snacks - Sticky notes (drawing/writing) | - Health is more than physical activity and diet. - Being able to pursue personal interests important for health. |
|  | 2  (2;2 h) | 6 people with ID  1 support worker  3 researchers | Leisure activity center | - Establish relation - Develop knowledge of app preferences:   - List your favorite apps.  - Play designer: Designing a health app concept. | - Joint meal, snacks - Sticky notes (drawing/writing) | - Doing things together with others is a strong drive for healthy behaviors. |
|  | 3  (3;2 h) | 6 people with ID  1 support worker  2 researchers | Leisure activity center | - Develop knowledge of app preferences:   - build on earlier ideas. | - Generated app ideas were visualized and grouped into clusters using a digital whiteboard. | - Important to build confidence through experiences of success, both in digital use and physical activity. - Wanting to clearly track one’s progress. - Simple design with minimal text, clear icons, and easy login. |
| **Group 2** | 4  (35;1.5 h) | 9 people with ID  3 support workers  4 researchers | Digital | - Establish relation - Describe the aim of the project - Develop knowledge of health: In what ways are you physically active? | - Spinner for turn taking | - Physical activity works best when integrated into existing routines, for example at the daily activity center. - Bound by what support workers can facilitate. |
|  | 5  (42;1.5 h) | 9 people with ID  5 support workers  3 researchers | Folk high school | - Develop knowledge of how involved one is in making dietary choices. - Develop knowledge in motivation for physical activity | - Image‑based support to structure conversations. - Small group discussions - Exergames | - Bound by what support workers can facilitate. - Gamification supports motivation, rather than focusing on physical activity itself. |
| **The multidisciplinary co-design team** | 6  (47;2 h) | 6 people with ID  2 support workers  1 professional  3 UX^b^ designers  3 researchers | Venue 1, University | - Establish relation - Develop knowledge of app preferences: List your favorite apps for well-being. - Ideas for a digital diary | - Joint meal, snacks - Sticky notes (drawing/writing) - Rapid ideation in small groups - digital diary - Voting with stickers | - Simple design with minimal text, clear icons, and easy login. - Track progress and activities over time. - Automatic or low‑effort registration of progress and activities. - Optional sharing with others. |
|  | 7  (49;2 h) | 5 people with ID  2 support workers  1 professional  3 UX designers  3 researchers | Venue 1, University | - Ideas for:   - supporting physical activity  - reminders  - motivation | - Joint meal, snacks - Rapid ideation in small groups – physical activity - Brain writing in small groups – reminders and motivation - Voting with stickers - Exergames | - Motivation through gamification, rewards, and tailored feedback. - Development of several concepts. - Encouraging reminders rather than controlling ones. |
|  | 8  (51;2 h) | 5 people with ID  1 professional  2 UX designers  3 researchers | Venue 1, University | - Develop knowledge of how involved one is in making dietary choices. - Ideas for supporting healthy diet | - Joint meal, snacks - Inspiration from apps related to food. - Rapid ideation in small groups – healthy diet - Voting with stickers | - Tired of prescriptive advice. - Challenging to know what and how much to eat. - Supportive food‑related advice without pressure. |
|  | 9  (92;1.5 h) | 3 people with ID  1 support worker  1 professional  1 UX designer  1 researcher | Venue 1, University | - Re-establish relation - Choose and build on 2 of the concepts developed in earlier workshops. | - Joint meal, snacks - Voting with stickers on former ideas for app concept. | - Goal achievement and feedback as motivators. - Winning ideas: *Walk to Paris* and *Murder mystery game*. - Discomfort with sharing information online. |
|  | 10  (94;1.5 h) | 4 people with ID  1 professional  1 researcher | Venue 1, University | - Building on the ideas from last workshop. | - Joint meal, snacks - AI to build on ideas and concepts. | - Gamification supports motivation and engagement. - Accessible and simple design (large text, text-to-speech). - Adjustable challenge levels. - Social features emphasizing collaboration over competition. - Encouragement over negative feedback |
|  | 11  (96;1.5 h) | 4 people with ID  1 professional  2 UX designers  1 researcher | Venue 1, University | - Learning how ideas are turned into an app. - Presenting the concept to UX designers. - Building on the ideas from last workshop. | - Joint meal, snacks - Using the murder mystery concept to explore general ideas for the app. | - Motivation through a mystery‑based game concept. - Game clues to build health knowledge. - Simple and clear design, one step at a time. - Engaging and visually appealing graphics. |
|  | 12  (98;1.5 h) | 4 people with ID  1 professional  2 UX designers  1 researcher | Venue 1, University | - Ideas on how healthy eating could be integrated into the game. | - Joint meal, snacks - Inspiration from apps related to food. | - Challenging finding the right motivation for changing eating habits. - Game clues to build health knowledge related to food. |
|  | 13  (100;1.5 h) | 3 people with ID  1 professional  2 UX designers  1 researcher | Venue 1, University | - Ideas to support motivation for physical activity | - Joint meal, snacks - Exergames | - Physical activity becomes secondary to a more fun, engaging goal. - Sound can support motivation, though adjustable and optional. - Adjustable levels of difficulty |
|  | 14  (102;1.5 h) | 3 people with ID  1 professional  2 UX designers  1 researcher | Venue 1, University | - What will motivate people to use the app. - Desired effects of the app. | - Joint meal, snacks - Exploring and discussing different visual scales for evaluation. | - Motivation driven by curiosity, enjoyment, and achievable play rather than demands. - A sense of achievement supports motivation and continued engagement. - Knowing that the app aims to improve one’s health supports engagement. - Challenges with evaluation scales, simple thumbs up/down best. |
|  | STUDY VISIT  (114;1 h) | 2 people with ID  1 professional  2 UX designers  1 researcher | Venue 2, University | - Familiarizing with a new setting including practical travel arrangements. | - Campus tour - Joint meal, snacks |  |
|  | 15  (117;2 h) | 4 people with ID  1 professional  2 UX designers  2 researchers | Venue 2, University | - Building on earlier ideas for social interaction, eating, and physical activity. | - Joint meal, snacks. - Ranking previous ideas using thumbs up/down scales. - Small‑group discussions followed by whole‑group reflection. | - Gamification as a way to support motivation for physical activity. - Supporting learning about healthy diet through quiz. - Safe interaction with others an important feature. - A sense of achievement without comparison to others. |
|  | 16  (120;2 h) | 3 people with ID  2 UX designers  2 researchers | Venue 2, University | - Exploring 4 UX‑sketched concepts based on insights. | - Joint meal, snacks. - Ranking concepts mock-ups using thumbs up/down scales. - Small‑group discussions followed by whole‑group reflection. | - Winning concept: *Weekly mystery*: collecting clues through movement. - Sustained engagement through progress tracking and collaboration with others. |
|  | 17  (125;2 h) | 4 people with ID  2 UX designers  2 researchers | Venue 2, University | - Building on the *Weekly mystery* concept, exploring ways to collect clues and progress in the game. | - Joint meal, snacks - Ranking multiple potential challenges for collecting clues based on previous workshop discussions using thumbs up/down scales. - Small‑group discussions followed by whole‑group reflection. | - Diverse challenges linked to healthy behaviors. - Easy to carry out immediately. - Feel achievable. - Motivation driven by obtaining clues, not the challenge itself. |

^a^ID: Intellectual disability

^b^UX: User experience
