# Supplementary material for: Co-Designing a Digital Intervention for Health Promotion for People With Intellectual Disabilities: Qualitative Study of Team Members’ Reflections
Source: J Med Internet Res. 2026 Oct 16;28:e105248. doi: 10.2196/105248 (PMC13722925; doi:10.2196/105248)
Supplement: Supplementary file 3 [file jmir_v28i1e105248_app3.docx]

# The Semi‑Structured Reflection Guide

## Reflection meetings 1-3 and 5

1. What was planned and what was the intention with this approach?
2. What actually happened during the workshops and why?
3. Why did the process unfold in this way, including adaptations and collaborative dynamics?
4. What do these reflections imply for regarding co‑design with people with intellectual disabilities?

Additional prompts (applied to varying degrees):

Decision-making

- How would you describe how decisions were made in the workshops?
- In what ways did different perspectives shape decisions?
- Describe how decision-making was enabled?

Power dynamics and collaboration

- How would you describe collaboration within the team?
- In what ways did roles and responsibilities take shape in practice?
- What seemed to support or challenge a collaborative and inclusive environment?

Accessibility

- How would you describe the accessibility of the activities in the workshops?
- What seemed to support understanding and engagement?

Adaptations

- What kinds of adaptations were made, and how did they differ from other design developments?
- How did these adaptations influence participation and interaction within the team?

## Reflection meeting 4

During the reflection meeting, go through the workshops in groups (1–3, 6–14, and 15–17), as these involved different settings and partly different groups of participants. When appliable, use ranking scale to support discussion.

1. This is from the … workshop we did (show pictures) – do you remember?
- What do you remember from that workshop?
- How was it for you to take part?
- How was it to work together with others in that group?
- What helped you, or made it harder?
- How was it to be in this place?
- What would you like to change?
  2. Would you like to tell me how you felt about…
- Eating together, the material we used, the time we had for the workshops, the information you received prior to workshops, working in small groups?

Important	Very good

Not important	Very bad
